# Effects of Nebivolol therapy on hemodynamic parameters and lipid profile compared to other beta blockers in patients with essential hypertension: a systematic review and meta-analysis

**DOI:** 10.22088/cjim.15.1.2

**Published:** 2024

**Authors:** I Made Fermi Wikananda, I Gusti Ngurah Metta Nurcahya, Putu Gede Pradipta Mahardika Wijaya, I Gde Raka Widiana, Dwijo Anargha Sindhughosa

**Affiliations:** 1 Faculty of Medicine, Udayana University, Denpasar, Indonesia; 2 Department of Internal Medicine Udayana University / Prof Dr IGNG Ngoerah Hospital, Denpasar, Indonesia

**Keywords:** Nebivolol, Beta blockers, Hemodynamic, Lipid profile, Hypertension

## Abstract

**Background:**

Besides being commonly used to treat high blood pressure, beta blockers are a family of drugs that are primarily used to regulate irregular cardiac rhythms. Nebivolol is a third generation of beta blockers, which is highly cardioselective, about three times as selective as bisoprolol. In this study, we aimed to evaluate Nebivolol's effectiveness and safety in comparison to other beta blockers.

**Methods:**

We searched the online databases PubMed, ScienceDirect, and Cochrane Library for relevant RCTs evaluating Nebivolol's effect on hypertension management. Relative risk (WRR) and weighted mean difference (WMD), with a 95% confidence interval (CI) were utilized to quantify the impact of nebivolol medication in the treatment of hypertension using a random effects model.

**Results:**

Twelve RCTs are included in the study, the patient numbers in every attempt ranged from 42-273 and 1456 patients in all were included in this review. Nebivolol does not significantly reduce SBP, DBP and HR compared to other beta blockers (WMD −0.57 mmHg, 95% CI [−1.55; 0.42 mmHg] p=0.12; WMD −0.27 mmHg, 95% CI [−1.36; 0.82 mmHg] p=0.63 ; WMD 0.10 BPM, 95% CI [−4.11;1.31 BPM] p=0.96, respectively). Patients treated with Nebivolol has significantly lower LDL-C (WMD -8.88 mg/dL, 95% CI [−15.28; -2.48 mg/dL] p=0.007) and significantly higher HDL-C (WMD 2.30 mg/dL, 95% CI [0.75; 3.84 mg/dL] p=0.004.

**Conclusions:**

According to this study's findings, nebivolol is well tolerated and decreases LDL-C. And higher HDL-C than other beta blocker agents. This review does not recommend nebivolol as first-line treatment in hypertension as Nebivolol does not significantly reduce blood pressure and HR of patients.

Hypertension, or an indication of high blood pressure is a continuously higher blood pressure in the arteries. As of 2019, approximately two billion adults or a third of the world population have hypertension (1), it is more frequent in man and more common with age. Hypertension increases risk for is chronic kidney disease, chemic heart disease, and heart failure. A family of drugs known as beta blockers, usually written β-blockers, is primarily used to treat irregular heartbeats. Despite no longer being the majority of patients' first choice for initial treatments, this drug is the one still widely utilized to manage high blood pressure. The endogenous catecholamines noradrenaline and adrenaline's receptors are blocked by beta blockers, a competitive antagonist. Some beta blockers block the activation of all types of β-adrenergic receptors, designated as ‘β1, β2, and β3. β1 adrenergic receptors’ are mostly found in the kidneys and the heart. β2 adrenergic receptor are mostly found in the lungs, liver, gastrointestinal tract, vascular smooth muscle, uterus, and skeletal muscle. β3 adrenergic receptors are mostly found in fat cells (2).

Epinephrine and norepinephrine stimulation of beta receptors results in a positive chronotropic and inotropic impact on the heart and enhances cardiac conduction velocity. Stimulation of β1 receptor causes renin release, stimulation of β2 causes the smooth muscles to relax, the skeletal muscles to tremble, and the liver's glycogenolysis to rise. Stimulations on those beta receptors are inhibited by beta blocker therapies (3). Stimulation of β3 receptors generates lipolysis. The effects of beta blockers on hypertension are anticipated, the mechanism involves the reduction of cardiac output die to negative inotropic effects (4). 

Adverse drug reactions caused by the utilized beta blockers include dyspnea, diarrhea, nausea, bronchospasm, bradycardia, hypotension, heart block, sexual dysfunction, erectile dysfunction and/or alteration of glucose and lipid metabolism. Orthostatic hypotension is correlated with β1 antagonist treatment. Adverse effect associated with β2 adrenergic receptor antagonist are: bronchospasm, and peripheral vasoconstriction. β2 inhibitors also associated with alteration of glucose and lipid metabolism. Adverse effects of β2 inhibition are rarer with β1 selective beta blockers (often called cardioselective beta blockers), but at higher doses, receptor selectivity diminishes (5). Nebivolol is a 3rd generation of beta blockers, which is highly cardioselective. In a experiment conducted, ff the beta blockers tested, nebivolol was identified to be the most β1 – selective; it was roughly three times more selective than bisoprolol (6), which is the 2nd generation of beta blockers. In humans, the drug's receptor selectivity is more complicated and is dose-dependent. Nebivolol is highly cardioselective at dosages of 5 mg (7); however, over 10 mg, it loses its cardioselectivity (7). 

It has been demonstrated that new generation beta-blockers are superior than conventional beta-blockers in terms of hemodynamic and metabolic parameters (1). In this study, we aimed to assess nebivolol's effectiveness and safety in comparison to other beta blockers. 

## Methods

**Search Strategies: **This systematic review was performed in compliance with PRISMA 2020 Guidelines8. We looked through the internet directories PubMed (Search conducted on December 2022), ScienceDirect (Search conducted on December 2022), and Cochrane Library (Search conducted on December 2022) for research assessing Nebivolol's effects on the management of hypertension. The entirety of our sourcing strategy for PubMed, Science Direct, and Cochrane library includes the following search string: “(“Hypertension” OR “High Blood Pressure”) AND “Nebivolol”. The search has been restricted to human studies. We also looked through the included papers' bibliographies. The literature search was carried out separately by three authors. Any differences of opinion were resolved through consensus or a fourth reviewer. This study has passed the ethical evaluation with the number of 765/UN14.2.2.VII.14/LT/2023.

**Inclusion criteria: **Studies might be included if they satisfied the following requirements: (1) study must be a Randomized Controlled Trial (RCTs); (2) population of the study must be adult patients with essential hypertension; (3) Intervention group was given Nebivolol 5mg; (4) Control group was given other Beta-blockers except for Nebivolol; (5) Duration of treatment is at least >2 weeks; (6) Outcomes were systolic and diastolic blood pressure reduction, heart rate reduction, serum LDL-C and HDL-C changes.

**Exclusion criteria: **Studies involving individuals with acute and chronic renal disorders and secondary hypertension were subject to the exclusion criteria. Studies were also disregarded if the entire patient population of <40 or if the complete text of the publication was unavailable.

**Quality assessment: **Utilizing the Jadad quality scale, which rates blinding, randomization, and accounting of all patients, the efficacy of each included trial was evaluated. Studies with scores of 0–2 are considered to be of poor quality, 3–4 are regarded as being of average quality, and 5 are regarded as being of the highest calibre.

**Data collection: **From the included articles, the following information was taken: study name, number of patients, patient primary diagnosis, year of publication, control treatment, intervention treatment, patient count for each therapy group, length of treatment, and the outcomes reported. In statistical analysis, intervention group were included if the treatment was Nebivolol 5mg monotherapy. The maximum prescribed dose that was available in any specific study was chosen for the control group. Each of the data's three writers independently extracted.

**Data analysis:** For continuous variables, baseline characteristics were summed together for each trial and provided as mean ‘±standard deviation’ and number (%) for categorical categories. Systolic and diastolic blood pressure (SBP and DBP, respectively), heart rate (HR), and changes in serum LDL-C and HDL-C were the outcome measures for the meta-analyses. The outcome for continuous data was reported as the mean difference. A standard χ^2^ test and I^2^ statistic were utilized to evaluate heterogeneity, with significance set at p <0.05 and I^2^ >50%, respectively. When there is high variability between studies and the findings are displayed utilizing forest plots, the random-effects model is used. Both random-effects and fixed-effects models were utilized if I^2^ < 50%. Only the random-effects model's findings are shown if both models produced similar results. Visual examination of funnel plots was used to evaluate publication bias, while Egger and Begg tests were employed to quantify it. All statistical analyses were carried out utilizing Review Manager 5.4, with the statistical significance level was set at p < 0.05 (Cochrane.org).

## Results

**Figure 1:**
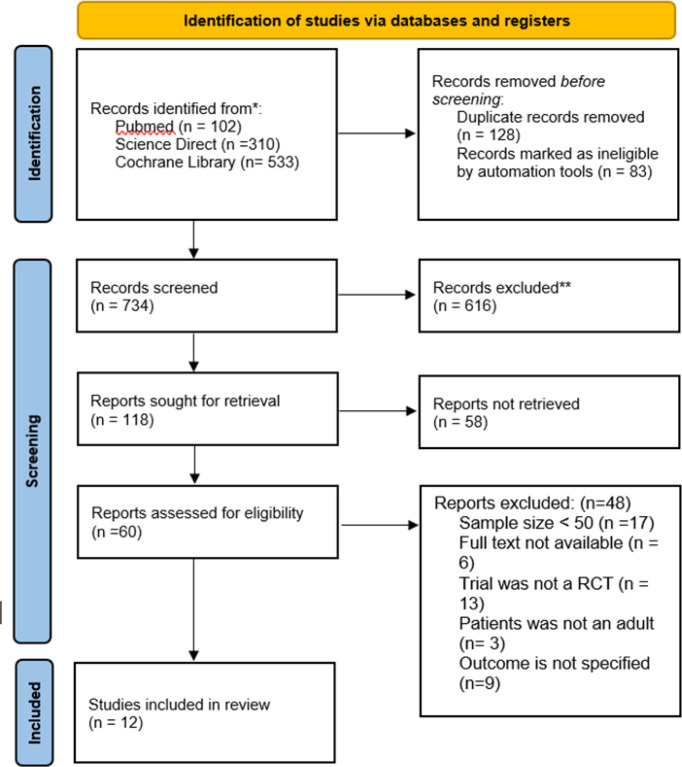
Flow diagram of the literature search and trial selection process

**Description of studies: **The initial string of searches in PubMed, Cochrane Library, and Science Direct produced 945 articles. 128 duplicates were removed, and 83 articles were marked as ineligible. 734 articles were reviewed by titles and abstracts, 118 possible suitable articles were discovered. 58 articles were unavailable for retrieval. 60 articles then assessed if they meet the inclusion and exclusion criteria. Of these, 12 articles met the criteria (figure 1). Characteristic of studies investigating BP reduction, HR reduction, LDL-C and HDL changes of Nebivolol monotherapy vs. other beta blockers are shown in table 1 and 2. Each experiment had between 42-273 people, and there were 1456 patients in all for this review. The trials ranged in length from from 2 weeks to 48 weeks.

**Figure 2:**
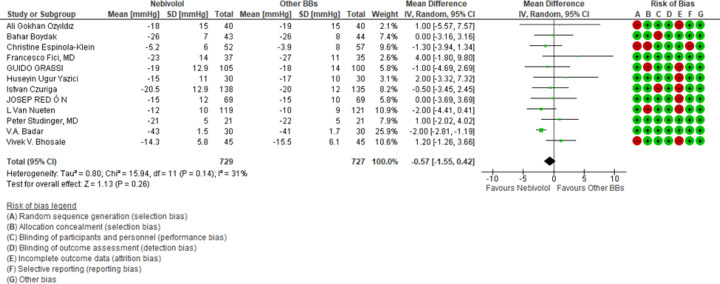
Effect of Nebivolol therapy compared to other BBs on Systolic BP reduction

**Figure 3:**
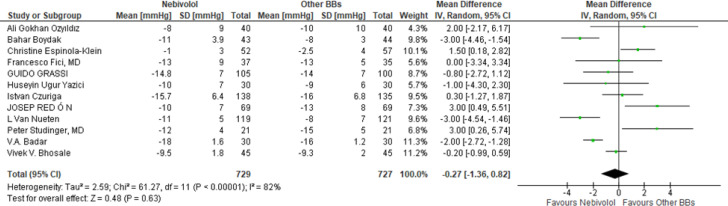
Effect of Nebivolol therapy compared to other BBs on Diastolic BP reduction

**Efficacy of Nebivolol on SBP and DBP reduction: **To evaluate its efficiency of Nebivolol vs. other BBs in reducing SBP and DBP, we analysed twelve RCTs^8-20^, with a total of 1456 patients. Nebivolol does not significantly reduce both SBP and DBP than other Beta-blockers (WMD −0.57 mmHg, 95% CI [−1.55; 0.42 mmHg] p=0.12 for Systolic BP seen on figure 2.); (WMD −0.27 mmHg, 95% CI [−1.36;0.82 mmHg] p=0.63 for Diastolic BP seen on figure 3).

**Table 1:** Characteristics of studies included in this study (9-20)

**Author**	**Year of Publication**	**Patients’ main diagnosis**	**Intervention**	**Control**	**Primary endpoint**	**Secondary endpoints**	**Duration**
Studinger et al	2013	Essential hypertension	Nebivolol 2.5 mg	Metoprolol 50 mg	SBP and DBP changes from baseline	Heart rate changes from baseline	12 weeks
Badar et al.	2011	Essential hypertension	Nebivolol 5 mg	Atenolol 50 mg	SBP changes from baseline	DBP, HR, and lipid profile changes	24 weeks
Nueten et al.	1998	Essential hypertension	Nebivolol 5 mg	Atenolol 50 mg	SBP changes from baseline	DBP and HR changes, adverse effects	4 weeks
Redon et al.	2014	Essential hypertension	Nebivolol 5 mg	Atenolol 50 mg	SBP changes from baseline	DBP and HR changes	10 weeks
Boydak et al.	2005	Essential hypertension	Nebivolol 5 mg	Atenolol 50 mg	SBP changes from baseline	DBP and HR changes, Sexual effects	12 weeks
Bhosale et al.	2014	Essential hypertension	Nebivolol 5 mg	Atenolol 50 mg	SBP changes from baseline	DBP, HR, Lipid profile, liver function, Hb, changes	12 weeks
Gokhan et al.	2016	Essential hypertension	Nebivolol 5 mg	Carvedilol 25 mg	SBP changes from baseline	DBP. HR. Lipid profile changes	16 weeks
Czuriga et al.	2003	Essential hypertension	Nebivolol 5 mg	Bisoprolol 5 mg	SBP changes from baseline	DBP and HR changes, adverse effects	12 weeks
Klein et al.	2011	Essential hypertension with PAD	Nebivolol 5 mg	Metoprolol 95 mg	Ankle Brachial index changes from baseline	SBP and DBP changes, adverse effects	48 weeks
Fici et al.	2013	Essential hypertension	Nebivolol 5 mg	Metoprolol 100 mg	SBP changes from baseline	DBP, HR, BSL, and lipid profile changes	2 weeks
Grassi et al.	2003	Essential hypertension	Nebivolol 5 mg	Atenolol 100 mg	SBP changes from baseline	DBP and HR changes, adverse effects	12 weeks
Yazici et al.	2013	Essential hypertension	Nebivolol 5 mg	Metoprolol 50 mg	SBP changes from baseline	DBP and HR changes	8 weeks

SBP: Systolic Blood Pressure, DBP: Diastolic Blood Pressure, HR: Heart Rate, Hb: Hemoglobin, BSL: Blood Sugar Level, PAD: Peripheral Artery Disease

The drugs investigated were: metoprolol, atenolol, bisoprolol, and carvedilol. Table 2 displays the findings from subgroup analysis in comparison to each of these drugs. Nebivolol has no significant reduction in SBP compared to metoprolol, atenolol, bisoprolol, and carvedilol (WMD -1.02 mmHg, 95% CI [-2.21 – 0.17mmHg], WMD 0.50 mmHg, 95% CI [-1.51 – 2.52mmHg], WMD -0.50 mmHg, 95% CI [-3.45 – 2.45mmHg], and WMD 1.00 mmHg, 95% CI [-5.57 – 7.57mmHg]), respectively. Nebivolol also has no significant reduction in DBP compared to atenolol, metoprolol, bisoprolol, and carvedilol (WMD -1.02 mmHg, 95% CI [-2.21 – 0.17mmHg], WMD 0.50 mmHg, 95% CI [-1.51 – 2.52mmHg], WMD -0.50 mmHg, 95% CI [-3.45 – 2.45mmHg], and WMD 1.00 mmHg, 95% CI [-5.57 – 7.57mmHg]), respectively. Full analysis can be seen on Table 2.

**Table 1:** Subgroup analysis of blood pressure reduction with Nebivolol therapy compared with other beta blockers

**agents**	**Trials**	**SBP (mmHg)**		**SBP (mmHg)**	
		**WMD**	**95% CI**	**WMD**	**95% CI**
**Atenolol**	6	-1.02	-2.21 – 0.17	-1.20	-2.51 – 0.10
**Metoprolol**	4	0.50	-1.51 – 2.52	1.21	-0.18 – 2.60
**Bisoprolol**	1	-0.50	-3.45 – 2.45	0.30	-1.27 – 1.87
**Carvedilol**	1	1.00	-5.57 – 7.57	2.00	-2.17 – 6.17

SBP: Systolic Blood Pressure

WMD: weighted mean difference

**Figure 4:**
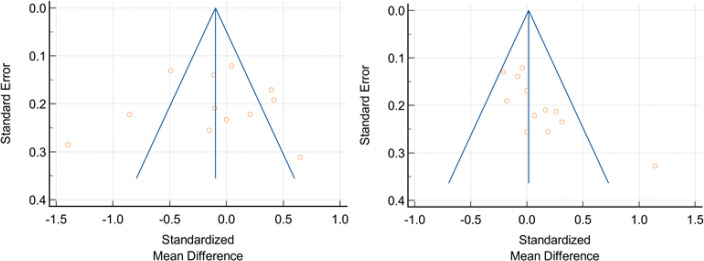
Funnel Plots of SBP dan DBP reduction Nebivolol compared to other BBs

**Publication Bias: **Publication bias measured using funnel plots as seen on figure 4. Interpreted quantitatively using Egger and Begg tests. Funnel plot on SBP reduction showed possibility of publication bias (Egger test: Intercept 3.4749; 95% CI [1.3681 to 5.5817]; p=0.0043). However funnel plot on DBP reduction showed no evidence of publication bias (Egger test: Intercept -0.4062; 95% CI [-6.1102 to 5.2978]; p=0.8771). Full analysis can be seen on Table 3. 

**Efficacy of Nebivolol on heart rate, LDL-C and HDL-C:**

**Figure 5:**
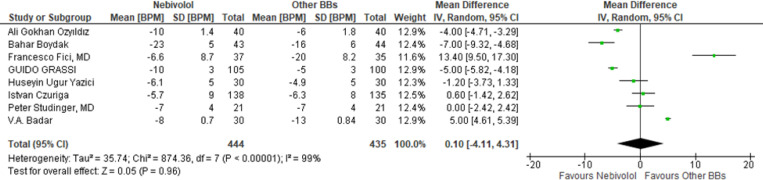
Effect of Nebivolol therapy compared to other BBs on HR reduction

To assess the efficacy of Nebivolol vs. other BBs in reducing heart rate, we analysed eight RCTs, 9,10,13,15,16,18,19,20 with a total of 879 patients. Nebivolol does not significantly reduce heart rate than other Beta-blockers (WMD 0.10 BPM, 95% CI [−4.11;1.31 BPM] p=0.96 for Heart rate seen on figure 5.) 

**Table 3:** Egger and Begg test on SBP and DBP reduction

	**Egger’s test**			**Begg’s Test**	
	**Intercept**	**95% CI**	**Sig.**	**Kendall’s Tau**	**Sig**
**SBP**	3.4749	1.3681 to 5.5817	0.0043	0.5758	0.0092
**DBP**	-0.4062	-6.1102 to 5.2978	0.8771	-0.1515	0.4929

**Figure 6:**
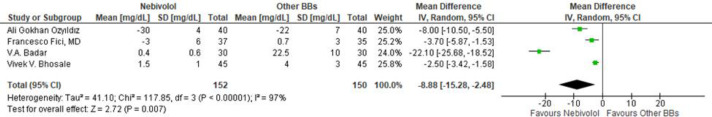
Effect of Nebivolol therapy compared to other BBs on serum LDL-C changes

**Figure 7:**
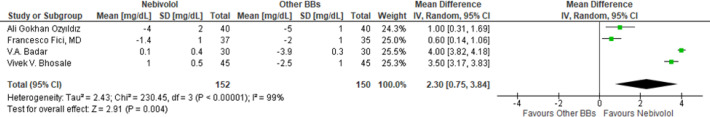
Effect of Nebivolol therapy compared to other BBs on serum HDL-C changes

We also analysed lipid profile of the included studies, among those studies, four RCTs compared the safety of nebivolol in terms of lipid profile, (10, 14, 15, 18) with a total of 302 patients. Patients treated with Nebivolol have significantly lower serum LDL-C profile (WMD -8.88 mg/dL, 95% CI [−15.28; -2.48 mg/dL] p=0.007). Patients treated with Nebivolol have significantly higher serum HDL-C profile (WMD 2.30 mg/dL, 95% CI [0.75; 3.84 mg/dL] p=0.004) LDL-C and HDL-C analyses can be seen on figure 6 and 7. 

## Discussion

**Efficacy: **This study demonstrates no discernible difference in the decrease of DBP, SBP, and blood pressure between nebivolol and earlier generations of b blockers. Nebivolol is a highly β1- selective third-generation blocker with endothelium-dependent vasodilatory effects that are mediated through the “L-arginine/NO pathway”, which has not been shown in other β blockers (such as atenolol and metoprolol) that are utilized in clinical practice. Bisoprolol and nebivolol exhibited comparable effects on the mean change in SBP and DBP in a previous trial, but there was no difference in overall effectiveness. The outcomes of a recent review by Jun-Ying Liu et al are in line with these findings (21).

Blood pressure drop depicted as a forest. WMD is a measure of the variation in blood pressure decrease between nebivolol-treated patients and the control group of patients who received treatment with other second-generation b blockers. Each individual study's point estimate of WMD is represented by a green square, and the square's size represents the study's weight. The point estimate and 95% confidence interval for every WMD are shown in black squares. SBP stands for systolic blood pressure, DBP for diastolic blood pressure, SD for standard deviation, IV for inverse variance, and 95% CI for 95% confidence interval; df, degrees of freedom; WMD, mean difference; Fixed, fixed effects model. According to another research, the difference in the mean reductions in SBP and DBP with Nebivolol failed to reach statistical significance and other beta blocker are meta-analysis by van Bortel. (22) and Ambrosioni (23).

Percentage of patients who responded to nebivolol as compared to other antihypertensive drugs. ARA = angiotensin receptor antagonists; ACEI = ACE inhibitors; BB = β-adrenoceptor antagonists; combined = all studies combined; CCA = calcium channel antagonists; control = antihypertensive drug utilized as comparator drug in that research; lower = lower limit of 95% CI of OR; OR = odds ratio; p-value = p-value of difference between nebivolol and control; upper = upper limit of 95% CI of OR Nebivolol and other b blockers have been the subject of several clinical trials as the management of hypertension has gained increasing attention. 

Meta-analyses are required to assess the efficacy and safety of nebivolol. Van et al carried out the first meta-analysis of nebivolol for hypertension (24). In contrast to this article, our study compares nebivolol against other beta blockers by using the most recent clinical studies on nebivolol.

Furthermore, we performed a subgroup analysis according to the type of beta blockers used and analysed more endpoints such as reduction of blood pressure, HR, LDL, HDL. Visual examination of funnel plots was carried out to evaluate publication bias, while Egger and Begg tests were employed to quantify it. Nebivolol and other beta blockers were examined in this meta-analysis for the first time, and we found no discernible difference between them in terms of lowering blood pressure, SBP, and DBP across all subgroups and across the entire investigation. However, the LDL was significantly lower and HDL was significantly higher in patients who used nebivolol compared with those who used other beta blockers 

More clinical evidence is required for confirmation since the high degree of heterogeneity in this finding and the sample size that small and it is still debatable whether nebivolol has a different effect on patients' heart rates than other b blockers. Nebivolol's tolerance was noticeably better than that of other second-generation beta blockers, and it was linked to a reduced risk of adverse events (AEs) than other second-generation beta blockers. 

According to the Egger test and the aforementioned sensitivity analysis, there was publication bias or no visible heterogeneity. Despite some limitations, the meta-analysis met the inclusion criteria and included all clinical data that was available. Additionally, the included clinical trials have average to excellent quality, and our meta-analysis's findings are trustworthy because they took into account publication bias and sensitivity. First, the power of our analysis was constrained by smaller sample size and smaller clinical trial numbers. Second, there may be some variability due to variations in patient clinical treatment, such as the type of second-generation b blockers used in the control group. 

To further investigate the effectiveness and safety in clinical practice, substantial RCTs with the same drug dosages and duration of treatment for Nebivolol and other medicines are required. In the future, when additional clinical studies are released, a thorough subgroup analysis can be performed. 

**Safety and tolerability: **Nebivolol is generally well tolerated. This study showed that Nebivolol produces significantly higher HDL-C and lower LDL-C compared to other beta blockers. This effect may be interpreted as additional effect on better lipid profile and able to produce potential lower risk of cardiovascular events. In patients with symptomatic illness, however, treatment-related adverse events could have less of an effect on quality of life. According to this meta-analysis, nebivolol had less side effects. The individual's benefit-risk ratio shall be taken into account while prescribing, just like with any therapy decision. In the SOLVD (Studies of Left Ventricular Dysfunction) study (25) of patients with heart failure, enalapril caused a larger percentage of patients to experience cough (5% vs. 2%) and a larger percentage of patients to stop taking the drug due to cough (p < 0.0001) in the treatment group compared to the placebo group. Because pulmonary edema-induced cough brought on by ACE inhibitor-induced cough predominated over heart failure in many cases shortly after treatment, the reported cough rates in this research were probable low. In contrast, cough caused by ACE inhibitors may not be as well tolerated and may have a negative effect on quality of life in individuals with asymptomatic hypertension. 

In a double-blind study (26), carvedilol and the ACE inhibitor enalapril both decreased blood pressure equally when quality-of-life issues in people with mild-to-moderate hypertension were taken into consideration, but enalapril significantly increased the incidence of cough (12% vs 0% with carvedilol; p < 0.001). Nebivolol's favorable tolerability profile in this analysis, when compared to other antihypertensive medications generally and to other β-blockers in particular, supports the findings of a recent meta-analysis, which found that nebivolol had greater tolerance in contrast to other cardioselective β-blockers, indicating that not all β-blockers are not all created equal. The current research backs with the findings of previous nebivolol quality-of-life studies, revealing no difference in overall wellbeing between nebivolol medication and placebo (24) and losartan (27). 

Specific aspects of life quality, like men's erectile function, are unaffected by nebivolol (28, 29) and sustained exercise performance in those who are physically active, (30, 31) which supports the idea that nebivolol is not a traditional β-blocker even more. Antihypertensive medications shall not only have strong antihypertensive efficacy but also have a consistent antihypertensive impact for the duration of the whole dose interval. The fact that amlodipine, a long-acting medication, compared to atenolol, has exhibited decreased mortality during therapy supports this opinion. The trough-to-peak ratio, or the ratio between the smallest and highest antihypertensive impact, is significant in this regard. Nebivolol 5 mg once daily has a strong antihypertensive impact, with a high trough-to-peak ratio of 89%.

This reasoning is in line with the low risk that clinical investigations have indicated. Patients who have significant side effects from first-line therapy can safely transfer to nebivolol therapy due to the drug's safety profile is high: 89%.This consideration is consistent with the low risk reported in clinical trials. Considering the safety profile of nebivolol, patients experiencing severe adverse effects with first-line treatment can safely switch to nebivolol therapy.

**Study limitations: **Despite some limitations, the meta-analysis met the inclusion criteria and included all clinical data that was available. Furthermore, according to the findings of publication bias analysis and the sensitivity, our meta-analysis results are trustworthy and the listed clinical studies are of intermediate to exceptional quality. 

First, the power of our analysis was constrained by the small sample size and small number of clinical trials. Second, there may be some heterogeneity due to variations in patient clinical treatment, such as the kind of β blockers utilized in the control group. Third, we compared SBP and DBP at weeks 8, 12, and 24 using subgroup analysis. Fourth, there is little evidence available concerning other drugs that were used with nebivolol. To deeper investigate the effectiveness and safety in clinical practice, substantial RCTs utilizing the same drug dosages and duration of treatment for nebivolol and other medicines are required. In the years to come, when additional clinical studies are released, a thorough subgroup analysis can be performed.

 In conclusion, this study revealed that nebivolol does not significantly reduce SBP, DBP, and HR compared to other beta blockers and Nebivolol produces significantly higher HDL-C and lower LDL-C compared to other beta blockers. This review shows that Nebivolol is as effective as other beta blockers in treating hypertension. In addition, Nebivolol produces better lower LDL and HDL which may lower the risk of other cardiovascular disease.

### Conflict of Interests:

No conflict of interests exists with regard to this study.

### Authors’ contribution:

IMFW: Study design, data acquisition, drafting and critical revision of the article for important intellectual content and study supervision. IGNMN: Study design, data acquisition, analysis and interpretation, drafting and critical revision of the article for important intellectual content. PGPMW: Study design, data acquisition, critical revision of the article for important intellectual content. IGRW: study design, analysis, interpretation, drafting of the article for important intellectual content. DAS: study design, analysis, interpretation, drafting of the article for important intellectual content. All authors approved the final version of the article. 
